# Feasibility and acceptability of unconditional cash transfers intervention among unemployed young adults with first-episode psychosis in South Africa: a pilot randomized controlled trial

**DOI:** 10.1080/00207411.2026.2650235

**Published:** 2026-04-25

**Authors:** Joyce Mlay, Lise Jamieson, Vuyokazi Ntlantsana, Neliswa Gcabashe, Thirusha Naidu, Busisiwe Siphumelele Bhengu, Lindokuhle Thela, Saeeda Paruk, Bonginkosi Chiliza, Jonathan K. Burns, Richard Lessells, Andrew Tomita

**Affiliations:** a Discipline of Public Health Medicine, School of Medicine, University of KwaZulu-Natal, Durban, South Africa; b Health Economics and HIV and AIDS Research Division, University of KwaZulu-Natal, Durban, South Africa; c Health Economics and Epidemiology Research Office, School of Clinical Medicine, Faculty of Health Sciences, University of the Witwatersrand, Johannesburg, South Africa; d South African Centre for Epidemiological Modelling and Analysis (SACEMA), Stellenbosch University, Stellenbosch, South Africa; e Discipline of Psychiatry, School of Medicine, University of KwaZulu-Natal, Durban, South Africa; f Discipline of nursing, School of Health Sciences, University of KwaZulu-Natal, Durban, South Africa; g Department of Innovation in Medical Education, Faculty of Medicine, University of Ottawa, Ottawa, Canada; h Department of Public Health and Primary Care, University of Cambridge, Cambridge, United Kingdom; i Faculty of Health and Life Sciences, University of Exeter, Exeter, United Kingdom; j Centre for the AIDS Programme of Research in South Africa, College of Health Sciences, University of KwaZulu-Natal, Durban, South Africa; k KwaZulu-Natal Research Innovation and Sequencing Platform (KRISP), College of Health Sciences, University of KwaZulu-Natal, Durban, South Africa; l Centre for Rural Health, School of Medicine, University of KwaZulu-Natal, Durban, South Africa

**Keywords:** Acceptability, feasibility, FEP, pilot, RCT, UCT

## Abstract

There is a lack of high-quality evidence on cash transfers as an intervention to support young people with first-episode psychosis (FEP) in Africa. To address this gap, we conducted a pilot randomized controlled trial (RCT) with a 3-month follow-up period to assess the feasibility and acceptability of an unconditional cash transfer (UCT) intervention among FEP. We enrolled 60 unemployed young adults (18–29years) with FEP from three public hospitals in KwaZulu-Natal, SA, and allocated participants into the intervention (*n* = 30) or the control arm (*n* = 30) using simple randomization. The intervention arm participants received a monthly transfer of R1,350 for three consecutive months. All 30 participants in the intervention arm completed the three-month follow-up, and 24 (80%) completed follow-up in the control arm. Although the effect of UCT on clinical and social outcomes is not the primary focus of this RCT, we observed significantly greater quality of life (p < 0.05) and medication adherence (p < 0.02) in the intervention group as compared to control group at the 3-month follow-up period. Although personal/social functioning challenge (p = 0.24) and cumulative relapses (p = 0.10) were greater in the control group, no statistically significant group differences were observed. Our study contributes the first evidence on UCT feasibility and acceptability for FEP.

## Introduction

Psychosis is a rare disorder that typically emerges as a first episode psychosis (FEP) in young adults 15–25 years old (Mackrell & Lavender, 2004). The condition is associated with loss of contact with reality, including symptoms such as delusions and hallucinations, and can be a temporary or long-term experience. First episode psychosis (FEP) can effectively be treated with appropriate medication and therapy, leading to symptom remission. Continued access to mental health care is essential for long-term management, preventing relapses and achieving full recovery (Doyle et al., 2014; Fridgen et al., 2013; Gearing et al., 2018). Disengagement (loss to follow-up) is a clinical challenge, with the findings of a systematic review reporting that five out of 10 patients with psychosis disengage from treatment, adolescents and young adults being at particularly high risk (Moore, 2018). A lack of transportation funds to attend scheduled appointment visits has been a structural barrier to engagement. In particular, young adults experiencing FEP often face socio-economic challenges, particularly due to limited skills and unemployment. These difficulties contribute to poverty, which significantly affects the long-term well-being of individuals with severe mental health conditions (Lund et al., 2010; Mills, 2015).

In KwaZulu-Natal (KZN), similar observations in our qualitative study exploration indicate that individual-level factors such as lack of transport and food significantly contribute to the risk of relapse among individuals with severe mental illness (Mlay et al., 2024). Also, youth unemployment affects approximately four out of 10 individuals in KZN, with 26% of youth coming from poorer households (De Lannoy & Mudiriza, 2019; Mngoma & Ayonrinde, 2023). We argue that the main barriers to accessing health services among the socioeconomically vulnerable populations are poverty and lack of financial resources. This includes the inability to afford the cost of transport, meaning that removing such financial impediments may improve the uptake of health services and care-seeking, thereby preventing a relapse (Taaffe et al., 2016; Wamoyi et al., 2020). (De Lannoy & Mudiriza, 2019; Mngoma & Ayonrinde, 2023).

There are several ways to support individuals with psychosis, including an early intervention programme for psychosis, which improves clinical outcomes and promotes long-term recovery by offering coordinated, multidisciplinary care (Ingoldsby, 2010). One promising approach targeting individuals with psychosis [though not necessarily FEP] in South Africa involves incorporating peer recovery groups (Asher et al., 2024). The solution to many poverty-driven challenges, lies in the provision of financial resources and cash transfer interventions is one of them. Cash transfer interventions are an effective mechanism for promoting service uptake and overcoming the structural barriers of poverty, thereby ensuring good health. The cash grant may take two forms: either a conditional cash transfer (CCT) or an unconditional cash transfer (UCT) (Baird et al., 2013; Prencipe et al., 2021). CCTs, such as those outlined by Marshall and Hill (2015) (e.g., tied to school attendance), come with specific conditions.

In contrast, UCTs involve payments without attached conditions or required actions (Handa et al., 2014). Unlike CCTs, UCTs do not impose specific requirements, offering a less stressful and burdensome approach (Yoshino et al., 2023). Both are recognized for their positive impact on mental health by addressing fundamental needs, such as housing, food security, transport, and communication, thereby contributing to an individual’s overall quality of life and dignity (Baird et al., 2013). Evidence from the systematic review shows that cash transfer interventions have proven effective in managing common mental disorders, such as anxiety and depression, but have not been used to address psychosis, including FEP (Pega et al., 2017; Pettifor et al., 2016).

Despite their potential benefits, a recent systematic review highlighted a lack of evidence regarding the ability of cash transfers to support socially vulnerable youth who have experienced FEP in sub-Saharan Africa (SSA), including in SA (Zimmerman et al., 2021). In response to this evidence gap, we conducted an open-label, two-arm pilot randomized trial named the Poverty Reduction Strategy for First-Episode Psychosis (PRS-FEP) trial to address the feasibility and acceptability of a UCT intervention following FEP among unemployed youth in rural KZN.

## Methods

### Study design and trial outcomes

The PRS-FEP is an open-label, two-arm pilot randomized control trial with a 3-month (12 weeks) follow-up period to assess the feasibility (recruitment rate, assessment completion, retention, and adoption) and acceptability (perception, appropriateness of the cash transfer method, and satisfaction) of the UCT intervention. Secondary outcomes included medication adherence, relapse, quality of life (QoL), and personal and social functions.

### Study setting and recruitment

This study was conducted in three public government hospital facilities in KZN between July 2023 and April 2024. The hospitals are located in Msunduzi municipality with a population of 679,000 people, 80% of whom are Black Africans; over half live in rural areas, and the unemployment rate is more than one in three people (Conroy et al., 2016). As a nested study within PSYchosis MAPping (PSYMAP-ZN) platform, the potential participants were referred from PSYMAP-ZN and approached for study participation (Mlay et al., 2022).

### Study population and sample size

We determined the sample size of 60 based on the guidelines for a pilot randomized controlled trial (Lancaster et al., 2004). The following inclusion criteria were applied: a) positive FEP status diagnosed by a psychiatrist under the ICD-11 criteria for psychotic disorders, b) in remission, c) on antipsychotic medication for <6 months after initial hospital discharge, d) age 18–29 years, e) isiZulu (language spoken to in the study area) or English speaking, f) unemployed, and g) resident in the Msunduzi municipality for ≥6 months. First episode psychosis is defined in this study as “the use of antipsychotic medication for less than six months after the initial psychiatric hospital visit” (Breitborde et al., 2009). Remission was defined as the absence of positive and negative symptoms at enrollment time or mild symptoms (2 or less positive or negative symptoms assessed by the research assistant). The exclusion criteria were: (h) absence of written consent, and i) lack of capacity to participate in the study.

### Data collection

A trained interviewer, fluent in English and isiZulu, informed potential study participants about the study after the PSYMAP-ZN research assistant team had referred them. All study participants were assessed at times T1 = enrollment/baseline, T2 = end of month 1, and T3 = end of month 3. The baseline (T1) interview data was collected at the hospital for outpatient and inpatient (those still admitted and awaiting discharge). The follow-up interview (T2 and T3) data were collected at the participants’ household post-discharge.

### Measurement

#### Primary outcomes

The data on feasibility and acceptability were collected at the three points to determine the average monthly recruitment, assessment completion, retention, adoption perception, satisfaction with the amount, and appropriateness of the cash transfer method of the UCT intervention (Table 1).

#### Clinical and social outcomes

Secondary endpoint of the pilot, included medication adherence, relapse, quality of life (QoL), and personal and social functioning. Medication adherence and relapse data were collected at two time points, while data on Quality of life and personal and social functioning were collected at three time points (Table 1).

1. Medication adherence was assessed using the Visual Analog Scale (VAS). VAS was self-administered and scored by the participants, allowing them to subjectively rate their level of medication adherence (Kalichman et al., 2009). We did not include validation by counting the number of pills left, as most participants reported that they were with their caretaker, and only a few consistently presented them for counting. Self-reported adherence was considered good for those scoring above 90%, based on previous studies that assessed medication adherence in antipsychotic medication (Lage & Hassan, 2009).
2. Relapse was measured using Cornell’s Service Index (CSI). CSI tracks the frequency and types of health services an individual utilizes over a defined period (e.g., the past month, three months, or six months), including inpatient psychiatric hospitalizations (Brodman et al., 1952).
3. Quality of life, which focuses on family relations, was evaluated using the Lehman Quality of Life Scale (LQoL). LQoL measured quality of life by assessing family relationships, which evaluated the objective and subjective family relationships (Liao et al., 2022; Thorup et al., 2010). The objective measurement of the frequency of family contact was determined by the average ratings on two aspects: (a) telephone conversations with a family member and (b) in-person meetings with a family member. Response options ranged from not at all, less than once a month, at least once a month, at least once a week, and at least once a day, scoring 1 through 5, respectively. The subjective satisfaction of the family relationships was based on the mean score of four items: (a) their overall family experience, (b) the frequency of family interactions, (c) the behavior of family members toward one another, and (d) the overall quality, these being rated from 1 = terrible to 7= delighted (Patrick et al., 2010). The Cronbach’s alpha for the frequency of contact and satisfaction measures ranged from 0.22 to 0.49 and 0.82 to 0.90 at baseline, four weeks, and 12 weeks, respectively.
4. Personal and social functioning was assessed using the Personal and Social Performance Scale (PSP). The PSP scale domains include socially useful activities, personal and social relationships, self-care, and disturbing and aggressive behavior. The total score was categorized based on the summary meaning score: 71–100 (only mild difficulties), 31–70 (varying degrees of disability), and 1–30 functioning poorly (Morosini et al., 2000). In addition to the above clinical data, the study collected participants’ socio-demographic information.

### Randomization

An independent statistician assisted with computer-generated numbers 1–60 based on a simple randomization method, and the allocations were placed in sealed, sequentially numbered envelopes. At enrollment, followed by the baseline assessment, the study principal investigator (PI) and research assistant opened the first envelope, followed by the numbers in sequence, and assigned each participant to the control or intervention arm. As this was an open-label study, the study clinic staff and the participants were not blinded to the allocation.

### Study intervention

All participants with FEP (in both the intervention and control arms) received the standard of care (SoC) as part of the routine health service, which included refilling antipsychotic medication prescriptions, planning the next visit, providing counseling services, where appropriate, and constant appointment reminders using short message service (SMS) or calls twice a week before a study visit. The participants received care from various health care providers, including a psychiatrist, general medical doctor, psychologist, nurse, or social worker. In addition, the UCT intervention arm participants received cash without attached conditions. The outpatients received R1,350 (US$90) based on the 2022 exchange rate at the end of the first interview. The inpatients received the same after they had been discharged, with two subsequent installments of the same amount at the end of months one and two (i.e., a total of R4,050 (US$270) for three months). We selected the amount of R1,350 (US$90) per month (adjusted for 2022 inflation, rounded up) as equivalent to what the South African Institute for Economic Justice (Saifaddin, 2021) has valued as the upper-bound poverty line. This amount was lower than the disability grant (R1890) but significantly higher than the social relief of distress grant R350 (US$23) (Mathebula et al., 2022). The PI and research assistant administered the cash payments to the participants at the hospital site (first payment), with subsequent payments being made either at home, at the hospital site, or at a convenient location. Both groups received R150(US$10) reimbursement per study visit for time, inconvenience, and travel expenses, in accordance with the South African National Ethics Guidelines (NDoH, 2024).

### Data analysis

We used descriptive statistics to summarize the baseline socio-demographic and clinical characteristics of the study participants in the intervention and control arms, including frequencies (%), means with standard deviation, and medians with interquartile range (IQR). We describe the adoption, retention, assessment completion, perception, appropriateness of the cash transfer method, and intervention satisfaction by calculating proportions for each measure within each arm and using the mean to determine the monthly recruitment rate. Second, although the design of the study is underpowered to reliably estimate the efficacy of the intervention against social and clinical outcomes, we conducted statistical comparisons between groups on medication adherence, relapse, quality of life, and personal and social functioning between T1 and T2, T1 and T3, and T2 and T3. We evaluated group clinical differences based on Chi-square, Fisher’s exact, or Wilcoxon rank-sum tests depending on whether the dependent variables are continuous/categorical and parametric/nonparametric. Third, we investigated social and clinical outcome comparison over time within group (i.e., intervention and control arm separately). Paired observations between time points within the same group of individuals were compared using McNemar’s paired test (categorical data) and signed-rank test (continuous data). We analyzed the data using STATA 18.

### Ethical considerations

The PRS-FEP study was approved by the Biomedical Research Ethics Committee of the University of KwaZulu-Natal (BREC,00004117/2022). The study has been approved by the KZN Department of Health (KZ_2002209_033) and registered with the South African National Clinical Trial Registry (#DOH-27-092022-5894). Written informed consent was obtained from all participants.

## Results

### Socio-demographic and clinical characteristics (n = 60)

**Figure 1:**
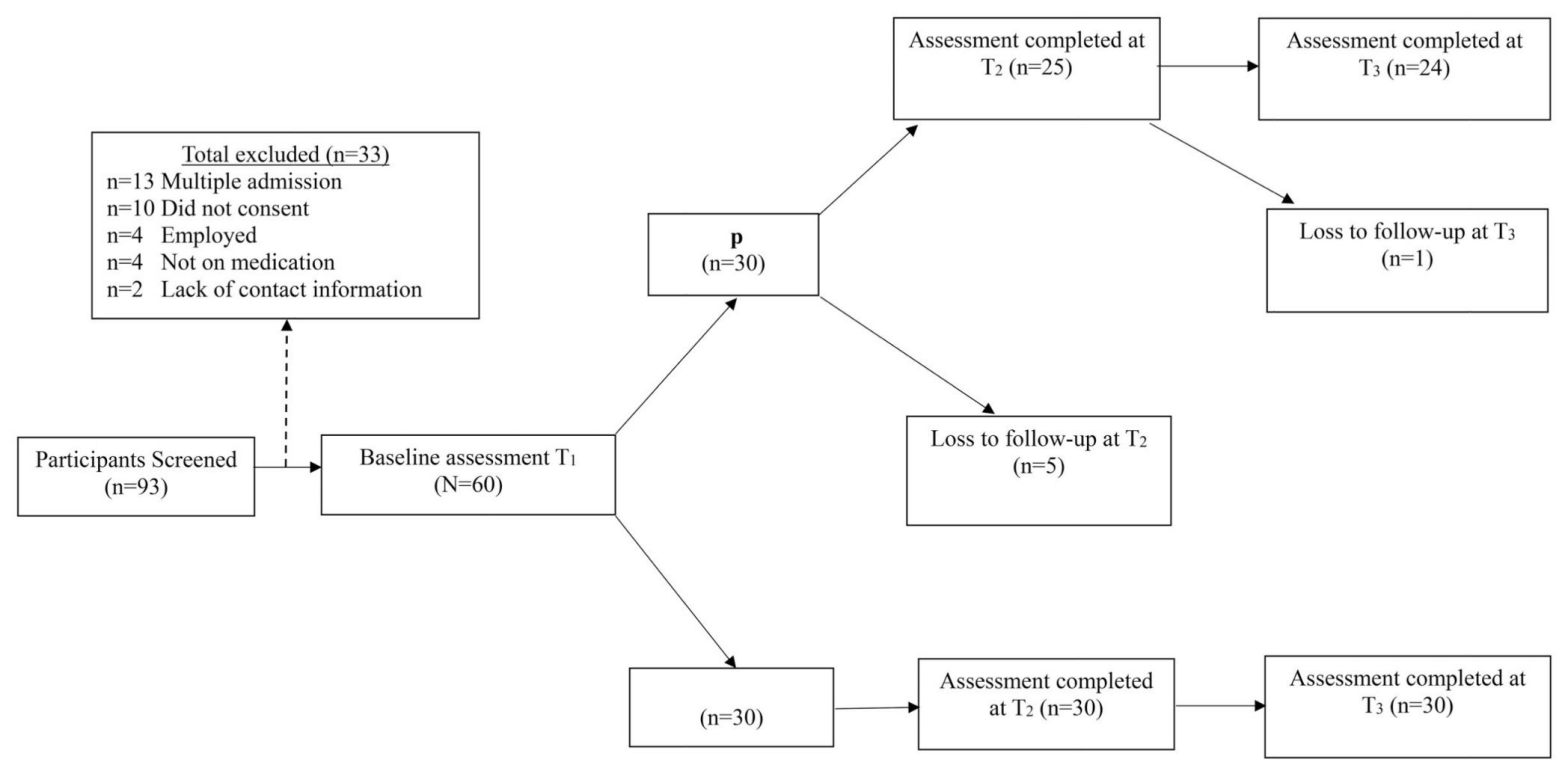
Participant selection and assessment process. T_1_=enrollment/baseline, T_2_=end of month 1, and T_3_=end of month 3

Of the 93 patients referred from the PSYMAP-ZN study, who were asked to provide written informed consent, 60 met the eligibility criteria and were enrolled (Figure 1). We enrolled 60 participants between July 2023 and December 2023, who were mostly male (*n* = 47, 78.3%), Black African (*n* = 58, 96.7%), with a median age of 23 years (IQR = 20–25.5). More than half had completed secondary school or had a bachelor’s degree (*n* = 34, 56.7%), and all were single, with the majority (*n =* 50, 83.3%) having no children. Approximately half (*n =* 28, 46.7%) resided in rural areas, and with most living with their families, 36 (60%) reported an average household income below the food poverty line of R624 per individual (Saifaddin, 2021). Half (*n =* 30, 50.0%) were diagnosed with schizophrenia, and three-fourths (*n =* 44, 73.3%) self-reported as being HIV-negative. Comparing baseline characteristics by intervention (*n =* 30) and control arm (*n =* 30) status, there were more male (90.0%% vs 66.7%%), poorer (73.3% vs 46.7%), self-reporting as HIV-negative (90%% vs 56.7%), and schizophrenia diagnosed participants (70.0% vs 30.0%) in the intervention arm (Table 2).

### Primary outcomes (feasibility and acceptability)

Feasibility (recruitment rate, retention, assessment completion, and adoption):

We recruited 10 participants per month, enrolling 60 young adults with FEP over six months. Of these, 54 (90%) completed the three-month follow-up period and the assessment: 30 (100%) from the intervention group, and 24 (80%) from the control group. All participants in the intervention group, having completed the three-month follow-up period, received R4,050 (US$270) during the study period. The participant flow chart is noted in Figure 1.

Acceptability (perception, satisfaction, appropriateness with the cash transfer methods):

All 60 participants perceived at enrollment that the UCT intervention would help them remain in care. In the intervention arm (*n =* 30), the level of satisfaction changed over time, with more than half (*n =* 19, 63.3%) being satisfied with the amount received after four weeks; this percentage declined after 12 weeks, with less than half remaining satisfied (*n =* 13, 43.3%) Two participants were dissatisfied with the cash transfer method and suggested bank transfers instead, without giving the reasons for their preference (Table 3).

### Clinical and social outcomes (medication adherence, relapses, quality of life, personal and social function)

#### Medication adherence

There were no differences in self-reported medication adherence between groups after four weeks of follow-up. At 12 weeks of follow-up, there was a significant difference in the medication adherence in the intervention group compared to the control group (73.3% vs. 41.7% with self-reported adherence >90% based on VAS, p = 0.02) (Table 3). Paired comparisons showed increased adherence within the intervention group (+6.6%) and decreased adherence in the control group (−18.3%) between four weeks (T2) and 12 weeks (T3), but neither change was statistically significant (Table 4).

#### Relapse

At four weeks of follow-up, the proportion of relapses was lower in the intervention group (3.3% *n =* 1) compared to the control group (16% *n =* 4); however, these differences were not statistically significant. We did not observe relapse at 12 weeks of follow-up in either group; all relapses occurred in the first four weeks of follow-up (Table 3).

#### Quality of life

At four weeks of follow-up, a difference in quality of life was measured objectively (median score 3.5 in intervention arm vs 3 in control arm, z=−2.485, p = 0.001) and subjectively (5.5 vs 4.8, z=−1.949, p = 0.05). Although the median scores remained higher in the intervention group at twelve weeks of follow-up, the differences were not statistically significant (Table 3). The paired comparison showed no change in the median score in the control group; the intervention group showed a slight improvement (+0.5) with no statistically significant difference observed for objective quality of life. The subjective median score in quality of life slightly declined at four weeks, followed by an improvement at 12 weeks in the control group, with no changes observed in the intervention group over the same period (Table 4).

#### Personal and social functioning

After four weeks, the degrees of disability improved from varying degrees to mild difficulties in both groups, with 100% (*n =* 0) improvement in the intervention group at baseline (*n =* 7) and 24.7% (*n =* 2) in the control group at baseline (*n =* 11). However, the difference was not statistically significant. At 12 weeks, a slight decline in social functioning was noted in 6.7% (*n =* 2) of the intervention group and 8.7% (*n =* 4) of the control group, but without a statistically significant difference (Table 3). Paired comparisons at four weeks revealed significant improvements in social functioning in both groups, which were statistically significant. At 12 weeks, although improvements were observed, they were not statistically significant (Table 4). No differences were found at baseline between the intervention and control groups (Table 2).

## Discussion

This pilot study assessed the feasibility and acceptability of the UCT intervention among unemployed young adults with FEP using a randomized controlled trial. The findings show that implementing the UCT intervention was feasible and well-accepted in this population. Although not the focus of the pilot trial, we observed significantly greater quality of family relationships (objective domain) and medication adherence results in the UCT group during the study. This pilot trial is one of the few UCT studies in SSA regions, targeting the FEP population.

On the topic of feasibility, we enrolled 60 participants from the PSYchosis MAPping in KZN (PSYMAP-ZN) platform. The trial target was set at 30 participants to be recruited monthly over a two-month period. In this trial, we recruited a mean of ten (10) participants per month, enrolling 60 over six months. The slower-than-anticipated recruitment was due to differences in the inclusion criteria between the two studies. Our study focused on young (18–29) and unemployed individuals, resulting in a smaller pool of eligible participants, as most participants enrolled in the PSYMAP-ZN were older than 29 years. Recruitment was confined to district, regional, and tertiary facilities. Future studies should consider expanding recruitment to primary healthcare clinics, as most patients with FEP continue to collect their medication from these sites.

Speaking to the point about feasibility (and its lessons learnt), this pilot RCT trial achieved an 30 (100%) retention rate in the intervention group and 24 (80%) retention rate in the control group at 12 weeks. Although the participants understood the concept of randomization during the informed consent process, there may have been some dissatisfaction as all those who declined follow-up belonged to the control arm, with one withdrawing due to a lack of interest. This finding aligns with earlier research, which indicated that many participants consented to join a study mainly due to their interest in being allocated to the intervention arm (Lindström et al., 2010). However, some participants were disappointed at being assigned to the control group, which may lead to withdrawal or refusal to participate in follow-up assessments, resulting in attrition bias (Nunan et al., 2018). Nonetheless, even in the control group that only received standard of care (SOC), it is a noteworthy that 24 (80%) completed follow-up assessment As this his is significantly higher compared to the South African observation study conducted in routine healthcare services for FEP in KZN, which reported a 56.7% retention rate over the 3 months (Maru et al., 2025).

Regarding acceptability, 100% of participants at enrollment felt that the UCT, in principle, would help them remain in care, but its adequacy requires further discussion. Other past studies of financial interventions pointed to their benefit to address structural barriers (Angeles et al., 2019; Handa et al., 2014; Prencipe et al., 2021), particularly the lack of financial resources to cater for transport and food (Mlay et al., 2024). More than half of the participants in our trial reported initial satisfaction with the amount received, possibly due to all of them being unemployed and the money playing an important role in enabling them to meet their immediate basic needs, especially a need for transport to the clinic, which will help the patients to remain engaged in care. However, there was some evidence that satisfaction declined over time. We suspect that satisfaction may be related to the level and length of UCT. Although the amount in this study is comparable to that given in other cash transfer interventions (Angeles et al., 2019; Greene et al., 2017; Prencipe et al., 2021) and corresponds to the upper-bound poverty line calculated at an individual level in South Africa (Saifaddin, 2021), the amount is not specifically designed for patients with psychosis. As a chronic illness, it is well-established that individuals with serious mental illness have multiple and complex needs that require long-term support (Tomita et al., 2016). Family is essential in the lives of those with serious mental illness (Tomita et al., 2014). Our separate qualitative inquiry (Mlay et al., 2025) reveals that a few participants were not satisfied with the amount and frequently shared the money with other household members, which may have contributed to the decreasing satisfaction over time. While in favor of the support in principle, our study participants may have developed greater expectations for long-term support.

### Study limitation

Small sample size is the major limitation of this pilot study, which is underpowered to estimate the preliminary efficacy of the intervention and insufficient to support causal inference analysis. In addition, a small sample size may have contributed to differences in socio-demographic and clinical characteristics (namely sex, HIV status, household income, and more cases of schizophrenia) between the intervention/control groups. However, the sample size was determined in accordance with guidelines for feasibility studies of pilot randomized controlled trials (Lancaster et al., 2004). Lastly, the length of pilot study was modest, and we were unable to investigate long-term benefit of the intervention.

### Study strength

Notwithstanding the above limitation, this is the first randomized evidence on the feasibility and acceptability of an unconditional cash transfer (UCT) intervention among unemployed young adults with first-episode psychosis (FEP) in South Africa.

### Future studies

The uptake of the UCT intervention was 100% by the 30 participants in the intervention arm, demonstrating potential for full adoption for future larger trials. Further evaluation of its real-world long-term effectiveness in a larger RCT may require a different approach. In addition to the suggestions above regarding future studies, the current intervention in this study was facilitated through direct cash transfers, which most participants preferred. This was manageable due to the small sample and short follow-up period, with different (such as discrete choice experiments) and larger studies needing to consider more secure automated distribution methods, such as bank or mobile phone transfers. Furthermore, although we observed significantly greater quality family relationships and medication adherence in the UCT group during the study, no significant difference was detected in relapse. It should be noted that one participant from the intervention arm experienced a relapse, while four participants from the control during the study. In light of findings about feasibility and acceptability, a fully powered trial may be warranted.

## Conclusion

This pilot provides novel and context-specific evidence that unconditional cash transfers are feasible, acceptable among young people with FEP, and potentially valuable for supporting mental health service engagement. The insights derived from this study can inform the development of a definitive randomized controlled trial, which will provide more robust evidence of the effectiveness of UCT as an intervention. In addition, the study demonstrates the need to enhance social protection mechanisms to support mental health treatment engagement for FEP, especially for unemployed young adults.

## Figures and Tables

**Table 1:** Assessment, evaluation schedule, and definition.

Outcome	Evaluation Domain	Evaluation Sub-Domain	Measures/Tools	Baseline interview		End of Month 1		End of Month 3		Baseline interview		End of Month 1		End of Month 3
				Intervention Group						Control Group				
				T_1_		T_2_		T_3_		T_1_		T_2_		T_3_
Primary trial outcome:	Feasibility	Adoption	Measured by obtaining the proportion of participants in the intervention arm who received three-round cash transfers during the study period.					X						
		Retention	Measured by obtaining the proportion of participants who completed three months of follow-up in the intervention and control arms.					X						X
		Assessment completion	Measured by obtaining the proportion of participants who complete the three-round interview assessment schedule visits in the intervention and control arms.					X						X
		Recruitment capability	Mean recruitment rate.	X						X				
	Acceptability	Perception	Measured at enrollment by obtaining the proportions of patients who think the cash transfer will help them engage in care.	X						X				
		Satisfaction	Measured by obtaining the proportion of participants who receive the cash transfer and are satisfied with it.			X		X						
		Appropriateness	Measured by the proportion of participants who agreed with the cash transfer methods.			X		X						
Clinical Outcome	Quality of life assessment (Family Relations)	Objective Subjective	The median score for Quality of Life using the Lehman Quality of Life (Family) objective and subjective measures.	X X		X X		X X		X X		X X		X X
	Personal and social functioning		Proportion of the participants with varying degrees of disability level using Personal and Social Performance Scale	X		X		X		X		X		X
	Relapse		Proportion of participants with readmission during the assessment period after hospital discharge using Cornel Service Index			X		X				X		X
	Medication adherence		Proportions of participants with good* psychiatric medication adherence assessed by visual analog scale over three month using the Visual Analog Scale			X		X				X		X

T_1_=enrollment/baseline, T_2_=end of month 1, and T_3_=end of month 3. although not mentioned, data on food (using Household food Insecurity access Scale) and water security (using individual water insecurity experiences [IWISe] scale were collected at baseline.

Good antipsychotic medication adherence is defined as 90%*(Lage & Hassan, 2009).

**Table 2:** Baseline characteristics by intervention status (n = 60).

Variable:	Groups	n	%	Intervention Group (%)	Control Group (%)	Degree	chi2	T-test	P-value	Exact
Gender:	Female	13	21.7	3 (10.0)	10 (33.3)	1	4.81		0.03	0.05
	Male	47	78.3	27 (90.0)	20 (66.7)					
Age:	Median (IQR)	23	(20-25.5)	23.5 (20-25)	23 (18-27)					0.99¶
Education:	Less than secondary school	26	43.3	15(50.0)	11(36.7)	1	1.09		0.30	
	Secondary school & Bachelor completed	34	56.7	15(50.0)	19(63.3)					
HIV status (self-reported):	Positive	6	10	1(3.3)	5(16.7)	2	8.53		0.01	0.01
	Negative	44	73.3	27(90.0)	17(56.7)					
	Unknown	10	16.7	2(6.7)	8(26.7)					
Race:	Black	58	96.7	28(93.3)	30(100.0)	1	2.06		0.15	0.49
	Nonblack	2	3.3	2(6.7)	0(0.0)					
Household average income:	Lower food poverty line	36	60	22(73.3)	14(46.7)					
	Food poverty line	6	20	1(3.3)	5(16.7)	3	9.42		0.05	0.04
	Lower bound poverty line	7	11.7	1(3.3)	6(20.0)					
	Upper or above upper bound poverty line	11	18.3	6(20.0)	5(16.7)					
Grant status:	Child support, social grants & COVID emergency grants	32	53.3	15(50.0)	17(56.7)	1	0.27		0.61	
	No grant	28	46.7	15(50.0)	13(43.3)					
Number of people living in a family:	Mean (SD)	5.6	(3)	5.2 (2.34)	6.1(3.45)	58		1.27	0.21	
Living status:	Living with the family	58	96.7	30(100.0)	28(93.3)	1	2.07		0.15	0.49
	Living alone	2	3.3	0(0.0)	2(6.7)					
Number of Children:	With no child	50	83.3	27(90.0.0)	23(76.7)	1	1.92		0.16	0.30
	At least one child	10	16.2	3(10.0.0)	7(23.3)					
Residence:	Urban & township	24	40	9(30.0)	15(50.0)	2	4.28		0.12	0.14
	Rural	28	46.7	18(60.0)	10(33.3)					
	Informal settlements	8	13.3	3(10.0)	5(16.7)					
Psychiatric diagnosis:	Schizophrenia	30	50	21(70.0)	9(30.0)	2	11.43		0.003	0.004
	Bipolar	9	15	1(11.1)	8(26.7)					
	Other psychosis	21	35	8(26.7)	13(43.3)					

¶ The age difference is based on the Wilcoxon rank-sum test.

**Table 3:** Feasibility, acceptability, and other clinical outcomes between groups.

			Baseline			1st Time Point (4 weeks)			Endline (12 weeks)		
Domain	Sub-Domain	Definition	Intervention (*n* = 30)	Control (*n* = 30)	Test statistics for comparison	Intervention (*n* = 30)	Control (*n* = 25)	Test statistics for comparison	Intervention (*n* = 30)	Control (*n* = 24)	Test statistics for comparison
1. Feasibility Outcomes:	1a. Adoption	Proportion of participants who qualified for UCT and successfully received three-round cash transfers during the study period.	N/A	N/A	N/A	N/A	N/A	N/A	30(100.0%)	N/A	
	1b. Retention	Proportion of participants who completed 3 months of follow-up in the intervention and control arms.	N/A	N/A	N/A	N/A	N/A	N/A	30(100.0%)	24(80.0%)	X^2^ = 6.56, p = 0.01
	1c. Assessment Completion	Proportion of participants who complete the interview assessment schedule visits within 3 months of follow-up in the intervention and control arms.	N/A	N/A	N/A	N/A	N/A	N/A	30(100.0%)	24(80.0%)	X^2^ = 6.56, p = 0.01
2. Acceptability Outcomes:	2a. Perception	Proportions of patients who think the cash transfer will improve disease outcomes at enrollment.	30(100.0%)	30(100.0%)	N/A	N/A	N/A	N/A	N/A	N/A	N/A
	2b. Satisfaction	Proportions of participants who received the cash transfer and were satisfied with it.	N/A	N/A	N/A	19(63.3%)	N/A	N/A	13(43.3%)	N/A	N/A
	2c. Appropriateness	Proportion of participants who agreed with the cash transfer methods.	N/A	N/A	N/A	29(96.7%)	N/A	N/A	28(93.3%)	N/A	N/A
3. Clinical Outcomes:	3a. Relapse	Proportion of participants with readmission during the assessment period after hospital discharge over three months.	N/A	N/A	N/A	1(3.3%)	4(16.0%)	X^2^=2.65, p = 0.10	0 (0.0%)	0 (0%)	N/A
	3b. Personal and social functioning	Proportion of the participants with varying degrees of disability level	7(23.3%)	11(36.7%)	X^2^=1.27, p = 0.26	0(0.0%)	2(8.0%)	X^2^=2.49, p = 0.11	2(6.7%)	4(16.7%)	_X_^2^=1.35, p = 0.24
	3c. Quality of life	The median score for Quality of Life using the scale (objective)	3(IQR2.5-5)	3(IQR2.5-4)	ranksum p = 0.72	3.50QR3-4)	3(IQR2-3)	ranksum p = 0.01	3.5(IQR3-4)	3(IQR2-4)	ranksum p = 0.46
		The median score for Quality of life using the scale (subjective)	5.5(IQR4-6)	5.1(IQR4-6)	ranksum p = 0.85	5.5(IQR4.8-6.3)	4.8(IQR4- 5.8)	ranksum p = 0.05	5.5(IQR4.8-6.3)	5.2(IQR4-6)	ranksum p = 0.27
	3d. Psychiatric medication adherence	Proportions of participants with good* psychiatric medication adherence were assessed using a visual analog scale over three months.	N/A	N/A	N/A	20(66.7%)	15(60.0%)	X^2^=0.22, p = 0.61	22(73.3%)	10(41.7%)	X^2^ = 5.54, p = 0.02

Average recruitment: 10 participants per month. df is 1 for all chi-square analyses. use of the exact test did not alter the significance of the findings. Good antipsychotic medication adherence is defined as 90% (Lage & Hassan, 2009).

**Table 4:** Social and clinical outcome comparison over time within group.

	Intervention Group		Test statistics for comparison	p-value	Control Group		Test statistics for comparison	p-value
	Baseline (*n* = 30)	1st Time Point (4 weeks, *n* = 30)			B aseline (*n* = 30)	1st Time Point (4 weeks, *n* = 25)		
Personal and social functioning	7 (23.3%)	0 (0.0%)	McNemar test	0.0001	11 (36.7%)	2 (8.0%)	McNemar test	0.02
Lehman Quality of Family Relations (Objective)	Median = 3(IQR2.5-5)	Median = 3.5(IQR3-4)	signed-test	0.07	Median = 3 (IQR2.5-4)	Median = 3 (IQR2-3)	Signed-test	0.48
Lehman Quality of Family Relations (Subjective)	Median = 5.5(IQR4-6) Baseline (n = 30)	Median =5.5(IQR4.8-6.3) Endline (12weeks, n = 30)	signed-test	0.13	Median = 5.1 (IQR4-6) Baseline (n = 30)	Median = 4.8 (4-5.8) Endline (12 weeks, n = 24)	Signed-test	1.000
Personal and social functioning	7 (23.3%)	2 (6.7%)	McNemar test	0.12	11 (36.7%)	4 (16.7%)	McNemar test	0.10
Lehman Quality of Family Relations (Objective)	Median = 3(IQR2.5-5)	Median = 3.5 (IQR3-4)	signed-test	0.42	Median = 3 (IQR2.5-4)	Median = 3 (IQR2-4)	signed-test	0.81
Lehman Quality of Family Relations (Subjective)	Median =5.5(IQR4-6)	Median = 5.5 (IQR4.8-6.3)	signed-test	0.23	Median = 5.1 (IQR4-6)	Median = 5.2 (IQR4-6)	signed-test	0.83
	1st Time Point (4 weeks, *n* = 30)	Endline (12 weeks, *n* = 30)			1st Time Point (4 weeks, *n* = 25)	Endline (12 weeks, *n* = 24)		
Adherence	20 (66.6%)	22 (73.2%)	McNemar test		15 (60.0%)	10 (41.7%)	McNemar test	0.17

Although not part of a formal analysis plan, personal and social functioning improved from baseline to 1^st^ time point among water secure group (p < 0.01). Similarly, personal and social functioning improved from 1^st^ time point to endline among the water secure group (p < 0.01). no improvement was observed in the water insecure group over time.
